# The non-structural protein μNS of piscine orthoreovirus (PRV) forms viral factory-like structures

**DOI:** 10.1186/s13567-015-0302-0

**Published:** 2016-01-08

**Authors:** Hanne Merethe Haatveit, Ingvild B. Nyman, Turhan Markussen, Øystein Wessel, Maria Krudtaa Dahle, Espen Rimstad

**Affiliations:** Department of Food Safety and Infectious Biology, Faculty of Veterinary Medicine and Biosciences, Norwegian University of Life Sciences, Postboks 8146 Dep, 0033 Oslo, Norway; Department of Parasitology, Norwegian Veterinary Institute, Postboks 750 Sentrum, 0106 Oslo, Norway; Department of Immunology, Norwegian Veterinary Institute, Postboks 750 Sentrum, 0106 Oslo, Norway

## Abstract

Piscine orthoreovirus (PRV) is associated with heart- and skeletal muscle inflammation in farmed Atlantic salmon. The virus is ubiquitous and found in both farmed and wild salmonid fish. It belongs to the family *Reoviridae*, closely related to the genus *Orthoreovirus*. The PRV genome comprises ten double-stranded RNA segments encoding at least eight structural and two non-structural proteins. Erythrocytes are the major target cells for PRV. Infected erythrocytes contain globular inclusions resembling viral factories; the putative site of viral replication. For the mammalian reovirus (MRV), the non-structural protein μNS is the primary organizer in factory formation. The analogous PRV protein was the focus of the present study. The subcellular location of PRV μNS and its co-localization with the PRV σNS, µ2 and λ1 proteins was investigated. We demonstrated that PRV μNS forms dense globular cytoplasmic inclusions in transfected fish cells, resembling the viral factories of MRV. In co-transfection experiments with μNS, the σNS, μ2 and λ1 proteins were recruited to the globular structures. The ability of μNS to recruit other PRV proteins into globular inclusions indicates that it is the main viral protein involved in viral factory formation and pivotal in early steps of viral assembly.

## Introduction

Piscine orthoreovirus (PRV) is a member of the family *Reoviridae*. The virus is associated with heart and skeletal muscle inflammation (HSMI), an important emerging disease in the intensive farming of Atlantic salmon (*Salmo salar*) [1, 2]. HSMI is mainly observed during the seawater grow-out phase and there is often a prolonged disease development [3]. The cumulative mortality varies from negligible to 20%, while the morbidity is almost 100% in affected cages [3]. PRV seems to be ubiquitous in Norwegian salmon farms [4]. Fish kept at high stocking density with frequent handling experience a stressful environment that may result in immunosuppression and a greater disease burden, thus facilitating the rapid spread of pathogens [5]. PRV has also been detected in wild salmon, but no lesions consistent with HSMI have been discovered in the wild population [6].

Phylogenetic analysis indicates that PRV branches off the common root of the genera *Orthoreovirus* and *Aquareovirus*, but most closely related to the orthoreoviruses [7, 8]. PRV differs from other orthoreoviruses like mammalian reoviruses (MRVs) and avian reoviruses (ARVs) in the ability to infect salmonid fish species at low temperatures, and in the preference for erythrocytes as one of the main target cells. The genome of PRV comprises ten double-stranded RNA (dsRNA) segments distributed in the classical orthoreoviral groups of three large, three medium and four small segments [1, 8, 9]. Currently, the PRV genome has been found to encode at least ten primary translation products. However, there is only a limited number of functional studies concerning the different proteins expressed by this virus [10, 11]. Based upon sequence homology to MRV, and the presence of conserved structures and motifs, eight of the deduced translation products are assumed structural components forming the orthoreovirus particle with an inner core and an outer capsid, while two of the translation products are non-structural proteins [8, 12].

A common feature for the non-structural proteins of reoviruses is their ability to form viral factories [13, 14]. Viral factories, also known as viroplasms or viral replication centers, are intracellular compartments for replication, packaging and assembly of viral particles [13, 15]. Several RNA and DNA viruses have been reported to induce these specialized membranous compartments within the cytoplasm of infected cells [16–18]. They commonly form as invaginations in a variety of organelles such as mitochondria, endoplasmic reticulum, lysosomes, peroxisomes, Golgi apparatus or chloroplasts [18, 19]. The factory scaffold facilitates spatial coordination of viral genome replication and assembly with the use of cell resources [18]. The viral factory inclusions seen during MRV infection consist of viral dsRNA, viral proteins, partially and fully assembled viral particles, microtubules and thinner filaments suggested to be intermediate structures [20]. Although organization of viral factories varies between different virus families, several fundamental similarities exist. Viruses utilize cellular biosynthetic pathways for their morphogenesis and propagation, and use a variety of mechanisms to avoid being wiped out by the cellular antiviral response [13, 21]. In the viral factories the viral pathogen-associated molecular patterns are shielded from inducing the activation of cellular innate responses [19].

Erythrocytes are major target cells for PRV, and in infected erythrocytes globular inclusions are formed and contain both PRV protein and dsRNA [22, 23]. The inclusions resemble the globular viral factories seen in MRV type 3 Dearing (T3D) prototype strain infected cells [19, 22]. Furthermore, the PRV inclusions contain reovirus-like particles as observed by transmission electron microscopy (TEM) [22]. This suggests that PRV, like MRV, forms viral factories in infected cells.

MRV μNS is the scaffolding protein that organizes viral factories during MRV infection [24]. Comparison of the PRV μNS amino acid sequence with the homologous proteins from MRV and ARV has revealed a very low sequence identity of only 17%, however, partially conserved motifs are present [8]. The latter includes a C-terminal motif shown for MRV μNS to be required for the recruitment of clathrin to viral factories [8, 25]. Furthermore, predictions of MRV and ARV μNS show two α-helical coils in their C-terminal region required for inclusion formation [26–29]. A high α-helical content in the C-terminal region is also predicted for the PRV μNS, but coiled coil motifs are predicted with significantly lower probability than for MRV and ARV [8]. In addition, MRV and ARV have both been shown to produce two protein products from gene segment M3 [8, 30]. Whereas μNS represents the full-length isoform, a second in-frame AUG (Met_41_) in the MRV protein represents the translational start site for the second isoform μNSC. In the ARV protein, post-translational cleavage near the N-terminal region creates μNSN [8, 30]. In PRV M3, only one open reading frame (ORF) has been identified encoding the μNS protein [8].

We hypothesized that the μNS of PRV is an organization center in the assembly of progeny virus particles. The aim in this study was to examine the localization of PRV μNS and its ability to interact with other PRV proteins in transfected cells.

## Materials and methods

### Cells

EPC cells (ATCC CRL-2872, *Epithelioma papulosum cyprini*) and CHSE-214 cells (ATCC CRL-1681, *Chinook salmon embryo*) were cultivated in Leibovitz-15 medium (L15, Life Technologies, Paisley, Scotland, UK) supplemented with 10% heat inactivated fetal bovine serum (FBS, Life technologies), 2 mM l-glutamine, 0.04 mM mercaptoethanol and 0.05 mg/mL gentamycin-sulphate (Life Technologies).

### Computer analyses

Multiple sequence alignments were performed using AlignX (Vector NTI Advance™ 11, Invitrogen, Carlsbad, CA, USA) and protein secondary structure predictions using PSIPRED v3.0. The presence of putative nuclear localization signals (NLS) in PRV μ2 was investigated using PSORTII, PredictProtein [31] and NLS mapper. The GenBank accession numbers for the PRV μNS, σNS, λ1 and μ2 coding sequences of the present study are KR337478, KR337481, KR337475 and KR337476, respectively.

### Plasmid constructs

**Figure 1:**
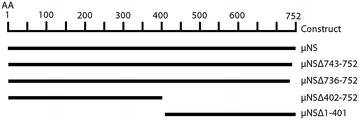
**Truncated μNS variants.** Schematic overview of the truncated μNS constructs.

Total RNA was isolated from homogenized tissue from a natural outbreak of HSMI in Atlantic salmon (MH-050607) as previously described [8]. RNA was denatured at 95 °C for 5 min and transcribed into cDNA using SuperScript^®^ III Reverse Transcriptase (RT) (Invitrogen) and Random Primers (Invitrogen) according to the manufacturer’s protocol. PfuUltra II Fusion HS DNA polymerase (Agilent, Santa Clara, CA, USA) was used to amplify the ORFs of μNS, σNS, μ2 and λ1. The primers contained the sequences encoding flag-tag, myc-tag or HA-tag for protein recognition by antibodies [32]. Primer sequences are shown in Table 1. For both the full-length μNS and σNS constructs, a pair of expression vectors was made encoding proteins tagged in either the C-terminus or the N-terminus; pcDNA3.1-μNS-N-FLAG, pcDNA3.1-μNS-C-FLAG, pcDNA3.1-σNS-N-MYC and pcDNA3.1-σNS-C-MYC. For μ2, the tag was added only C-terminally and for λ1 only N-terminally, pcDNA3.1-μ2-C-MYC and pcDNA3.1-λ1-N-HA, respectively. Four truncated forms of the μNS protein with flag-tags C- or N-terminally depending on the truncation were also generated to determine sequence regions in PRV μNS involved in formation of viral factories during infection, pcDNA3.1-μNSΔ1-401, pcDNA3.1-μNSΔ402-752, pcDNA3.1-μNSΔ736-752 and pcDNA3.1-μNSΔ743-752 (Figure 1). In-fusion HD Cloning Kit (Clontech Laboratories, Mountain View, CA, USA) was used to clone PCR products into the XbaI restriction site of the eukaryotic expression vector pcDNA3.1(+) (Invitrogen). Sanger sequencing (GATC Biotech AG, Konstanz, Germany) verified all sequences. A pcDNA3.1 construct expressing the protein encoded by infectious salmon anemia virus (ISAV) segment 8 open reading frame 2 (S8ORF2) protein [33] was used as a control during transfections, immunoprecipitation and western blotting.

**Table 1:** Expression plasmids.

Plasmid name	Primer	Sequence (5ʹ → 3ʹ)
pcDNA3.1-μNS-N-FLAG	Forward	GCCGCTCGAGTCTAGAGCCACC**ATG** *GACTACAAAGACGATGACGACAAG*ATGGCTGAATCAATTACTTTTG
	Reverse	AAACGGGCCCTCTAGATCAGCCACGTAGCACATTATTCAC
pcDNA3.1-μNS-C-FLAG	Forward	GCCGCTCGAGTCTAGAGCCACC**ATG**CGCAAGCTGGACTTGGTTGCA
	Reverse	AAACGGGCCCTCTAGATCA*CTTGTCGTCATCGTCTTTGTAGTC*GCCACGTAGCACATTATTCACGCC
pcDNA3.1-σNS-N-MYC	Forward	GCCGCTCGAGTCTAGAGCCACC**ATG** *GAACAAAAACTCATCTCAGAAGAGGATCTG*ATGTCGAACTTTGATCTTGG
	Reverse	AAACGGGCCCTCTAGACTAACAAAACATGGCCATGA
pcDNA3.1-σNS-C-MYC	Forward	GCCGCTCGAGTCTAGAGCCACC**ATG**TCGAACTTTGATCTTGG
	Reverse	AAACGGGCCCTCTAGACTA*CAGATCCTCTTCTGAGATGAGTTTTTGTTC*ACAAAACATGGCCATGATGC
pcDNA3.1-μ2-C-HA	Forward	GGCGGCCGCTCGAGTCTAGA**ATG**CCTATCATAAACCTGCC
	Reverse	GTTTAAACGGGCCCTCTAGA*AGCGTAATCTGGAACATCGTATGGGTA*CTCACCAGCTGTAGACCACC
pcDNA3.1- λ1-N-HA	Forward	CGCTCGAGTCTAGAGCCACC**ATG** *TACCCATACGATGTTCCAGATTACGC*TATGGAGCGACTTAAGAGGAAAG
	Reverse	AAACGGGCCCTCTAGATTAGTTGAGTACAGGATGAG
pcDNA3.1-μNSΔ743-753	Forward	GCCGCTCGAGTCTAGAGCCACC**ATG** *GACTACAAAGACGATGACGACAAG*ATGGCTGAATCAATTACTTTTG
	Reverse	AAACGGGCCCTCTAGATCACCAGTCATCTGAGCCACCAAA
pcDNA3.1-μNSΔ736-752	Forward	GCCGCTCGAGTCTAGAGCCACC**ATG** *GACTACAAAGACGATGACGACAAG*ATGGCTGAATCAATTACTTTTG
	Reverse	AAACGGGCCCTCTAGATCAGTCGATGATTTTTGGAAACTC
pcDNA3.1-μNSΔ1-401	Forward	GCCGCTCGAGTCTAGAGCCACC**ATG**CCAACCACCTGGTATTCAAC
	Reverse	AAACGGGCCCTCTAGATCA*CTTGTCGTCATCGTCTTTGTAGTC*GCCACGTAGCACATTATTCACGCC
pcDNA3.1-μNSΔ402-752	Forward	GCCGCTCGAGTCTAGAGCCACC**ATG** *GACTACAAAGACGATGACGACAAG*ATGGCTGAATCAATTACTTTTG
	Reverse	AAACGGGCCCTCTAGATCATGTGGTCAGGGAATAGTGCAT

Primers used in generating the constructs encoding PRV μNS (M3), σNS (S3), μ2 (M1) and λ1 (L3) and truncated versions of μNS.

Start codons are marked in bold and epitope tags in italic.

### Transfections of fish cells

EPC and CHSE cells were seeded on gelatin embedded cover slips (12 mm) with pre-equilibrated L-15 growth medium at a density of 1.5 × 10^4^ cells in a 24-well plate 24 h prior to transfection. Plasmids were transfected using Lipofectamine LTX reagent (Life Technologies) according to the manufacturer’s instructions. In brief, 2 μL lipofectamine was mixed with 0.5 μg plasmid and 0.5 μL PLUS reagent, and diluted in a total of 100 μL Opti-MEM (Life Technologies). After 5 min of incubation, the mixture was added to the cells and incubated at 20 °C for 48 h. When co-transfections were performed, a total of 0.4 μg of each plasmid were used and the amount of PLUS reagent was increased to 0.8 μL.

### Immunofluorescence microscopy

Transfected EPC and CHSE cells were fixed and stained using an intracellular Fixation and Permeabilization Buffer (eBioscience, San Diego, CA, USA). The cells were washed in Dulbecco’s PBS (DPBS) with sodium azide. Intracellular fixation buffer was added before incubation with primary (1:1000) and secondary antibodies (1:400) diluted in permeabilization buffer according to the manufacturer’s protocol. Antibodies against flag (mouse anti-flag antibody) and HA (rabbit anti-HA antibody) were obtained from Sigma-Aldrich (St Louis, MO, USA), while antibodies against the myc epitope (goat anti-myc antibody) was obtained from Abcam (Cambridge, UK). Secondary antibodies against mouse immunoglobulin G (IgG), goat IgG and rabbit IgG were conjugated with either Alexa Fluor 488 or 594 obtained from Molecular Probes (Life Technologies). Hoechst trihydrochloride trihydrate (Life Technologies) was used for nuclear staining. The cover slips were mounted onto glass slides using Fluoroshield (Sigma-Aldrich) and prepared for microscopy as described above. Images were captured on an inverted fluorescence microscope (Olympus IX81) and on a confocal laser scanning microscope (Zeiss LSM 710).

### Immunoprecipitation

A total of 5 million EPC cells were pelleted by centrifugation, resuspended in 100 μL Ingenio Electroporation Solution (Mirus, Madison, WI, USA) and co-transfected with 8 μg plasmid using the Amaxa T-20 program. pcDNA3.1-μNS-N-FLAG was co-transfected with pcDNA3.1-σNS-N-MYC, pcDNA3.1-μ2-C-HA, pcDNA3.1-λ1-N-HA and pcDNA3.1 S8ORF2 (negative control) separately, using three parallel preparations. The transfected cells were transferred to 75 cm^2^ culture flasks containing 20 mL pre-equilibrated L-15 growth medium (described above). From each culture flask, 0.5 mL transfected cells were transferred to a 24-well plate intended for expression analysis by immunofluorescence microscopy. Cells were collected from the culture flasks 72 h post transfection (hpt), centrifuged at 5000 *g* for 5 min and resuspended in 1 mL Nonidet-P40 lysis buffer (1% NP-40, 50 mM Tris–HCl pH 8.0, 150 mM NaCl, 2 mM EDTA) containing Complete ultra mini protease inhibitor cocktail (Roche, Mannheim, Germany). The mix was incubated on ice for 30 min, and then centrifuged at 9700 *g* for 12 min at 4 °C. The supernatant was transferred to a new tube, added antibodies against the desired epitope tag or anti-S8ORF2 and incubated overnight at 4 °C with rotation. The Immunoprecipitation Kit Dynabeads Protein G (Novex, Life Technologies) was used for protein extraction and the beads prepared according to the manufacturer’s protocol. The cell-lysate-antibody mixture was mixed with the protein G coated beads and incubated 2 h at 4 °C. The beads-antibody-protein complex was washed according to the manufacturer’s protocol.

### Western blotting

The beads-antibody-protein complex was diluted in Sample Buffer (Bio-Rad, Hercules, CA, USA) and Reducing Agent (Bio-Rad), denatured for 5 min at 95 °C and run in sodium dodecyl sulfate–polyacrylamide gel electrophoresis (SDS-PAGE), using 4-12% Bis–Tris Criterion XT gel (Bio-Rad). Lysates from non-transfected EPC cells were used as a negative control, and Precision Plus Protein Western C Standards (Bio-Rad) as a molecular size marker. Following SDS-PAGE, the proteins were blotted onto a polyvinylidene fluoride (PVDF) membrane (Bio-Rad) and incubated with primary antibody (anti-flag 1:1000) at 4 °C overnight. After incubation with secondary antibody (Anti-mouse IgG-HRP, GE Healthcare, Buchinghamshire, UK), the proteins were detected by chemiluminescense using Amersham ECL Prime Western Blotting Detection Reagent (GE Healthcare).

## Results

### Prediction of secondary structure

**Figure 2:**
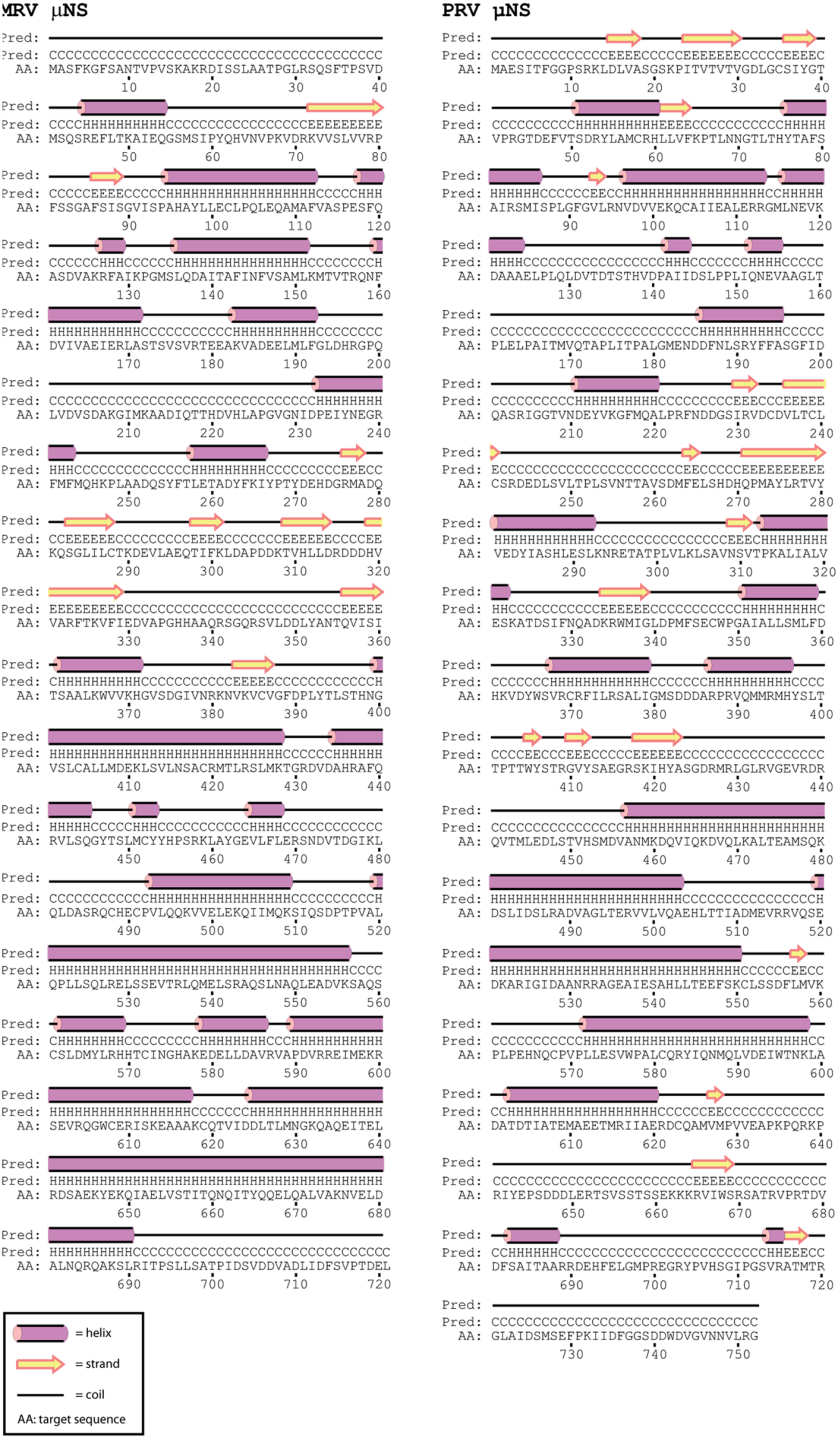
**Secondary structure predictions.** Secondary structure predictions of the μNS proteins from PRV and MRV (PSIPRED). Accession numbers for the MRV and PRV proteins are NC004281 and KR337478, respectively.

The predicted secondary structure profiles of PRV and MRV μNS were similar despite low sequence identity (Figure 2). The PRV μNS sequence in this study differs by twenty-three nucleotides of which twenty are silent (not shown) to that analyzed in a previous study (GU994018) [8]. The three amino acids that differed between the two PRV μNS sequences did not cause significant changes to the predicted secondary structures as determined by the PSIPRED program. The remaining three nucleotides all result in synonymous amino acid differences, i.e., displaying similar physiochemical properties (M/L_94_, I/V_451_ and A/V_498_). For σNS, the difference is six nucleotides and for λ1 twenty-eight, all silent. For μ2, the difference is fifteen nucleotides, all silent except for one synonymous substitution (R/K_113_).

### μNS forms viral factory-like structures

**Figure 3:**
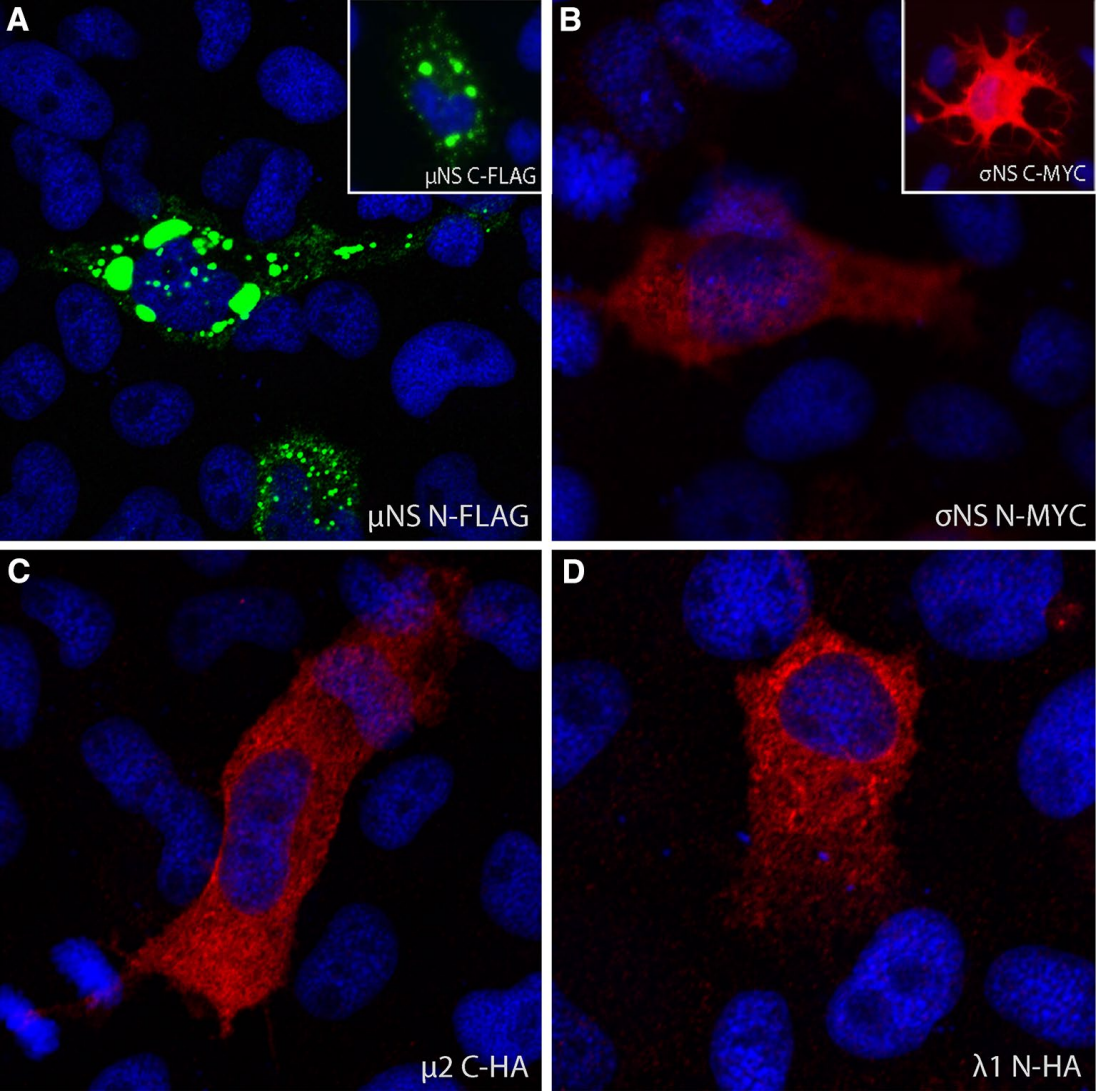
**Subcellular localization of PRV proteins.** EPC cells transfected with four different PRV plasmid constructs (µNS, σNS, λ1, µ2) processed for fluorescence microscopy 48 hpt. **A** EPC cells expressing μNS N-FLAG. Boxed region in top left corner shows EPC cells expressing μNS-C-FLAG. **B** EPC cells expressing σNS N-MYC. Boxed region shows σNS-C-MYC. **C** EPC cells expressing μ2-C-HA. **D** EPC cells expressing λ1-N-HA.

EPC cells transfected with pcDNA3.1-μNS-N-FLAG 48 hpt showed small, dense globular inclusions evenly distributed in the cytoplasm with some larger perinuclear inclusions 48 hpt (Figure 3A). A similar staining pattern was seen with the corresponding C-terminally flag-labelled construct (Figure 3A, insert), and in CHSE cells (not shown). EPC cells transfected with the σNS-N-MYC, μ2-C-HA or λ1-N-HA constructs were also examined 48 hpt (Figure 3B–D). The σNS-N-MYC protein was evenly distributed in the cytoplasm possibly with some minor nuclear localization (Figure 3B). A nucleocytoplasmic distribution pattern was also observed with the C-terminally myc-labelled σNS (Figure 3B, insert). Both the μ2-C-HA and λ1-N-HA proteins were evenly distributed in the cytoplasm (Figure 3C and D), with the former showing minor staining in the nucleus of some cells (not shown). Non-transfected cells did not show any staining (not shown).

### σNS, λ1 and μ2 are recruited to viral factory-like structures

**Figure 4:**
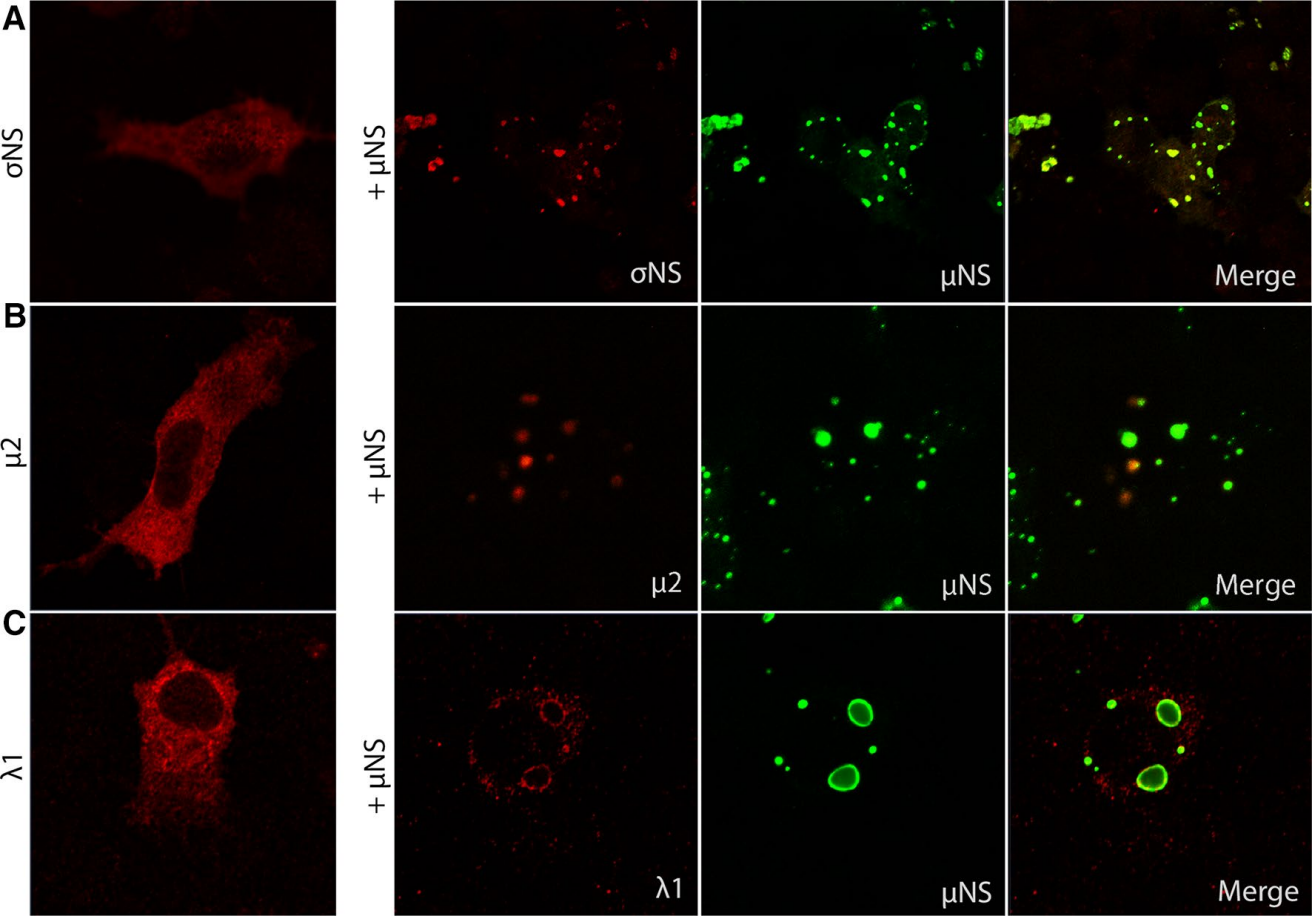
**Co-transfections with μNS.** EPC cells transfected with constructs encoding σNS, μ2 and λ1 and co-transfected with µNS. The cells were processed for confocal microscopy 48 hpt. **A** EPC cells transfected with σNS alone and cotransfected with μNS. **B** EPC cells transfected with μ2 alone and cotransfected with μNS. **C** EPC cells transfected with λ1 alone and cotransfected with μNS.

Viral proteins interacting with μNS were identified by co-transfecting EPC cells with pcDNA3.1-μNS-N-FLAG and separately with each of the σNS-N-MYC, μ2-C-HA or λ1-N-HA constructs. The μNS protein retained its globular distribution pattern in the presence of the other PRV proteins 48 hpt (Figure 4). In contrast, the staining pattern for σNS, μ2 and λ1 proteins changed from an evenly cytoplasmic distribution to globular inclusions co-localizing wholly or partially with the μNS protein (Figure 4A–C). Co-localization with μNS was most pronounced for σNS, and σNS was no longer found in the nucleus (Figure 4A). For μ2, the change in distribution was not as pronounced as for σNS and λ1, but in some cells μ2 formed small punctuated structures partially overlapping with the μNS globular inclusions (Figure 4B). Co-expression of σNS-N-MYC with either μ2-C-HA or λ1-N-HA, i.e. in the absence of μNS, did not alter staining patterns, and the viral factory-like structures were not formed (not shown).

### σNS and μ2 interact with μNS

**Figure 5:**
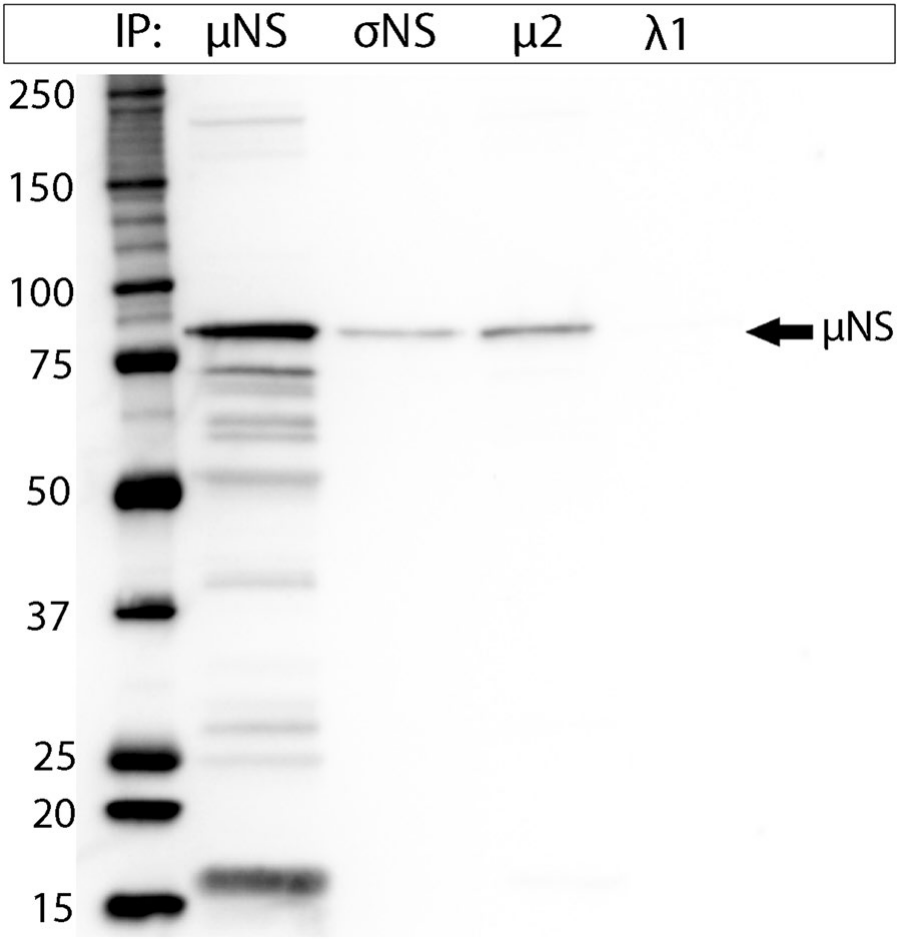
**Western blot of immunoprecipitated PRV proteins.** Lysates from EPC cells transfected with µNS alone or µNS together with σNS, μ2 or λ1 were used for immunoprecipitation (IP) targeting the different protein tags. Their ability to co-precipitate µNS was assessed by western blotting targeting µNS (84.5 kDa).

Immunoprecipitation and western blotting were performed to confirm interactions between PRV μNS and each of σNS, λ1 and μ2 (Figure 5). EPC cells were co-transfected with μNS-N-FLAG and separately with the σNS-N-MYC, λ1-N-HA and μ2-C-HA constructs. The results confirmed that μNS interacts with σNS and μ2. Interaction with λ1 on the other hand (Figure 5), or to the negative control ISAV-S8ORF2 protein, was not observed (not shown).

### Truncated μNS proteins

**Figure 6:**
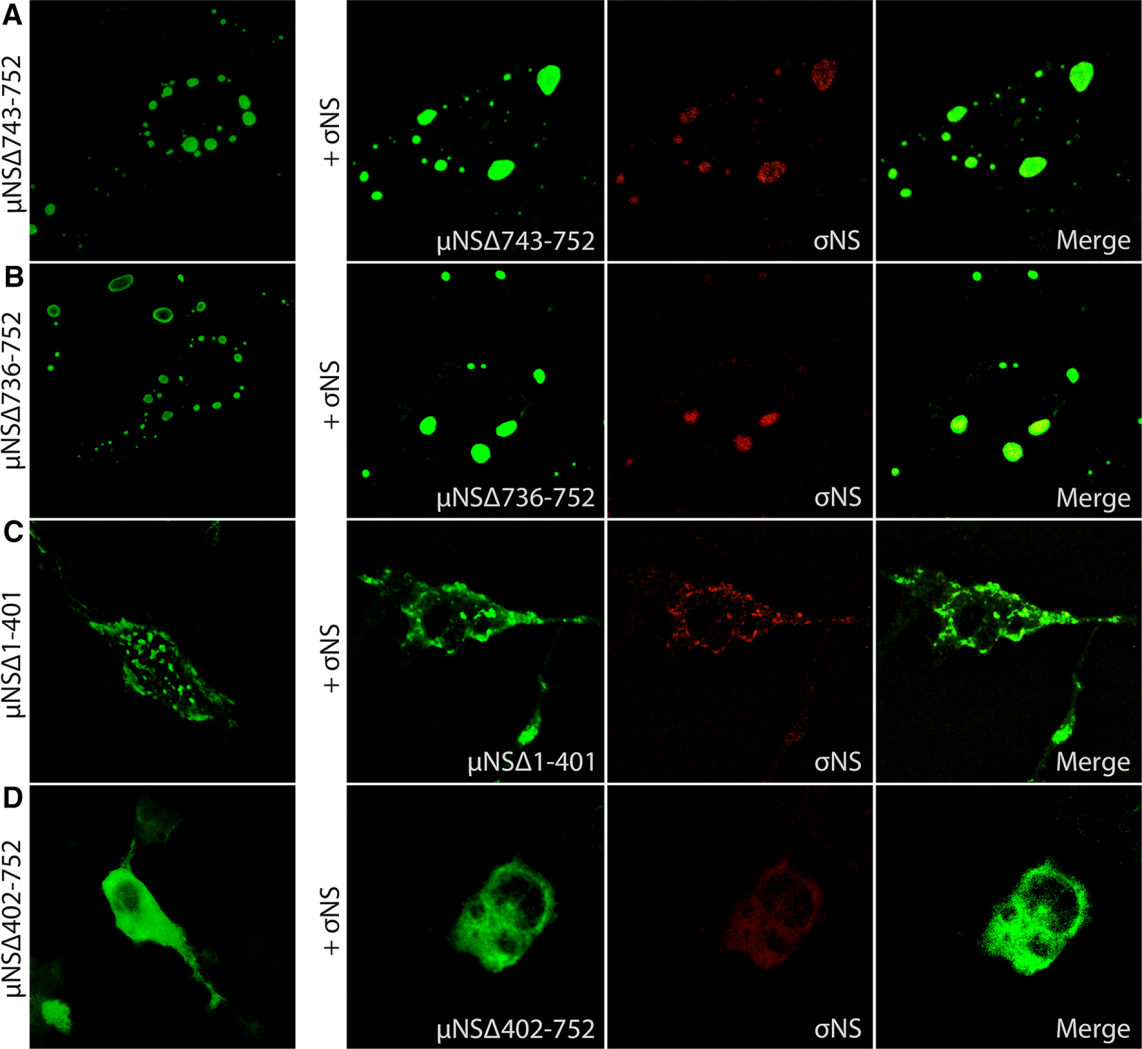
**Co-transfections with truncated μNS variants.** EPC cells transfected with pcDNA3.1-μNS-Δ743-752, pcDNA3.1-μNS-Δ736-752, pcDNA3.1-μNS-Δ1-401 and pcDNA3.1-μNS-Δ402-752 processed for fluorescence microscopy 48 hpt. **A** EPC cells expressing μNSΔ743-752 alone and co-expressed with σNS. **B** EPC cells expressing μNSΔ736-752 alone and co-expressed with σNS. **C** EPC cells expressing μNSΔ402-752 alone and co-expressed with σNS. **D** EPC cells expressing μNSΔ1-401 alone and co-expressed with σNS.

EPC cells were transfected with plasmid constructs encoding the truncated μNS variants μNS-Δ743-752, μNS-Δ736-752, μNS-Δ1-401 and μNS-Δ402-752 (Figure 1). Small, factory-like globular inclusions were formed by μNSΔ743-752 and μNSΔ736-752 (Figure 6A and B). Individual co-expression of these μNS truncated variants with σNS-N-MYC recruited the latter protein to the factory-like inclusions, similar to that observed with full-length μNS (Figures 4A, 6A and B). The μNSΔ1-401 protein formed small dense irregular or granular structures in the cytoplasm with reminiscences to the globular structures formed by the full-length protein (Figure 6C). The μNSΔ1-401 truncated version did also recruit and change the distribution pattern of σNS (Figures 3B and 6C). In contrast, μNSΔ402-752 was evenly distributed in the cytoplasm, and did not form viral factory-like structures. When μNSΔ402-752 was expressed together with σNS, both proteins were evenly dispersed throughout the cytoplasm (Figure 6D).

## Discussion

The reoviral factories are the sites for virus replication and particle assembly [19]. The MRV μNS is the scaffolding protein organizing the viral factories including gathering of core proteins, while the σNS protein facilitates construction of core particles and subsequent particle assembly [20, 24, 29, 34]. Viral factory-like structures have been observed in PRV infected Atlantic salmon erythrocytes in both in vivo and ex vivo experiments [22, 23]. In this study we demonstrated that PRV μNS alone forms dense globular, viral factory-like cytoplasmic inclusions. The globular, cytoplasmic distribution of μNS was not seen for the non-structural σNS or the structural μ2 and λ1 PRV proteins. However, these proteins changed their distribution pattern and co-localized with μNS in the dense globular structures when they were co-transfected with μNS. Co-transfection of σNS with μ2 or λ1 did not cause changes in distribution pattern. Expression of the N-terminal 401 amino acids did not form viral factory-like structures, mapping this feature to the remaining C-terminal 351 amino acids. Immunoprecipitation and subsequent Western blot analysis confirmed the association between μNS-σNS and μNS-μ2. Our findings strongly suggests that μNS is the prime organizer of viral factories for PRV.

MRV strains exhibit differences in viral inclusion morphology. Reovirus type 1 Lang (T1L) forms filamentous inclusions, whereas type 3 Dearing (T3D) forms punctate or globular inclusions [20, 35]. These morphologic differences are determined by the ability of the virus to interact with the microtubule system, a feature mapped to MRV μ2 [35]. In the filamentous factories, μ2 co-localize with and stabilize microtubules when expressed in cells in the absence of other viral proteins [20, 35]. PRV inclusions appear similar to the globular inclusion type, closely resembling the μNS-containing globular viral factories in reovirus T3D infected cells [35]. We cannot exclude that there are strains of PRV that forms filamentous inclusions. There might be several not yet recognized PRV-like viruses that infect other salmonid fish species. It has been proposed that the larger surface area of filamentous inclusions allow for more efficient viral replication through better access to small-molecule substrates or newly synthesized proteins from the surrounding cytosol [35]. Immunofluorescence and confocal microscopy have been used to identify globular and filamentous inclusions after transfection with expression plasmids encoding proteins from MRV and ARV [27, 36, 37].

Viral factories commonly form early in reovirus infection as small punctate structures throughout the cytoplasm that increase in size and become more perinuclear during infection [20]. The factories recruit viral proteins, which allow the efficient assembly of virus core particles [34, 38]. We observed that PRV μNS guided the σNS, μ2 and λ1 proteins to the viral factories. Our rationale for choosing σNS, μ2 and λ1 as co-transfectants was that these are examples of non-structural (σNS) and structural (μ2 and λ1) proteins in the core particle. MRV μNS and σNS are found in the first detectable viral protein-RNA complexes in MRV infected cells and form cytoplasmic inclusions similar to the viral factory-like structures formed in the absence of viral infection [36]. Analysis of MRV μNS transfected cells revealed that at 6 hpt, μNS inclusions were uniformly small and spread throughout the cytoplasm, whereas at 18 hpt and 36 hpt, larger perinuclear inclusions were present along with smaller inclusions [20]. In addition to its association with σNS, MRV μNS has been shown to interact with each of the five structural proteins that make up the core particle (λ1, λ2, λ3, σ2 and μ2) [24, 34]. Although it generally occurs within 18 hpt, strong co-localization between MRV μNS and the core surface proteins have been observed as soon as 6 h post infection [34]. Since PRV replicates at lower temperatures than MRV, the process of assembling core proteins to viral factories occurs at a slower rate. Studies on the ARV have identified a similar role of μNS in forming viral factories [27].

The nature of the globular inclusions and their interactions with other PRV proteins might differ in erythrocytes and established cell lines. However, neither cell line nor C– or N-terminal epitope tagging influenced the formation of dense globular structures by the PRV μNS. Transfection of salmon erythrocytes was not successful (data not shown). Still, globular-type inclusions are common in naturally PRV infected erythrocytes. This indicates that the formation of globular inclusion structures is an intrinsic property of μNS.

The ability of μNS to redirect the subcellular localizations of other PRV proteins can be mediated through protein–protein interactions. This was observed for σNS and μ2 following immunoprecipitation and western blotting. Many cellular proteins are only functional when localized to specific cellular compartments, and translocation to the appropriate sites can serve to regulate protein function [36]. Reovirus proteins involved in replication are only active within functional centers characterized by a particular location and protein composition [36]. We could not demonstrate protein–protein interaction between μNS and λ1, although confocal imaging clearly proved redistribution of λ1 when the protein was co-expressed with μNS. Interaction(s) between μNS and λ1 is therefore likely but perhaps through the involvement of a third cellular protein. Alternatively, the binding affinities between the two proteins are below the threshold detectable by the conditions used in the immunoprecipitation- and western blot assays. Further investigations are needed to study the mechanisms involved in λ1 redistribution when co-expressed with μNS. Since μNS expressed alone forms viral factory-like inclusions, and is responsible for the redistribution of other PRV proteins, it is likely one of the first proteins involved in virus factory formation and thereby essential in the early steps of viral replication.

Staining of σNS, and to some extend μ2, was observed in the nucleus of transfected cells. The size of the σNS protein, predicted to be 39.1 kDa, may allow passive diffusion through the nuclear pores, whereas the 86 kDa μ2 protein exceeds the 40 kDa limit for passive diffusion [39]. MRV σNS and μ2 are both shown to be distributed in the nucleus and the cytoplasm of transfected and infected cells. The ability of MRV σNS to locate in the nucleus of infected cells has been linked to its nucleic acid binding capability, while the presence of MRV μ2 in the nucleus of transfected cells is explained by predicted nuclear import and export signals [20, 24, 40–42]. There are no predicted classical nuclear localization signals (NLSs) in PRV σNS [8] or PRV μ2 (present study, using PSORTII and NLS mapper). The presence of nuclear export signals (NES) have though been predicted for both proteins. Neither σNS nor μ2 was found in the nucleus after co-transfection with μNS. As μNS does not localize to the nucleus, an explanation might be that μNS sequesters σNS and μ2 within the cytoplasmic inclusions, thus reducing the amount of free σNS and μ2 to enter the nucleus. This has also been proposed for MRV σNS and μ2 [20, 40]. Further studies are needed to excavate the functional roles of the observed nuclear localization of PRV σNS and μ2.

The C-terminal part of MRV μNS contains four distinct regions comprising 250 amino acids that are sufficient to form viral factories [29]. These regions include two predicted coiled-coil domains, a linker region between the coiled coils containing a putative zinc hook, and a short C-terminal tail [24]. PRV μNS may contain a coiled-coil motif in its C-terminal region [8]. A deletion of the eight C-terminal amino acids of MRV μNS results in diffusely distributed protein throughout the cytoplasm and the nucleus, suggesting that these amino acids are necessary for inclusion formation [29]. PRV μNS also contains a high α-helical content in its C-terminal region although the sequence identity to the homologous MRV protein is low [8]. In fact, the predicted secondary structure profiles of MRV and PRV μNS show significant similarities, highlighting the importance of conserving structural features over primary sequence for the function of homologues proteins across evolutionary lines. Still, the two C-terminally truncated forms of μNS containing deletions of 10 and 17 amino acids, respectively, formed viral factory-like structures when expressed in EPC cells, indicating that factory formation is not dependent on these amino acids. Deletion of the 401 N-terminal amino acids seemed to have some influence on the viral factory formation, but the protein still accumulated in granular structures and retained its ability to recruit σNS. Deletions of the 351 C-terminal amino acids, on the other hand, resulted in diffusely distributed protein and absence of globular inclusions. This indicates that the C-terminal region of μNS is essential for factory formation. The N-terminal region of PRV μNS displays a somewhat higher level of secondary structure conservation when compared to MRV. In MRV, this region of μNS is crucial for interactions with σNS, μ2, λ1 and λ2 [34, 38].

In conclusion, our results strongly suggest that PRV µNS protein is essential for factory formation and assembly of viral proteins, similar to that of μNS of other orthoreoviruses. Further studies on both the structural and functional properties of PRV proteins can provide important information relating to disease development following PRV infections.
